# Metabolomic and Transcriptomic Analysis Reveals Flavonoid-Mediated Regulation of Seed Antioxidant Properties in Peanut Seed Vigor

**DOI:** 10.3390/antiox13121497

**Published:** 2024-12-08

**Authors:** Fangping Gong, Di Cao, Zhuo Li, Yi Fan, Yaru Zhang, Lin Zhang, Kai Zhao, Ding Qiu, Zhongfeng Li, Rui Ren, Xingli Ma, Xingguo Zhang, Kunkun Zhao, Dongmei Yin

**Affiliations:** 1 College of Agronomy & Peanut Functional Genome and Molecular Breeding Engineering, Henan Agricultural University, Zhengzhou 450002, China; gongfangping@henau.edu.cn (F.G.); caod01@126.com (D.C.);; 2 Institute of Crop Germplasm Resources, Henan Academy of Agricultural Sciences, Zhengzhou 450002, China

**Keywords:** peanut, large seed, flavonoid, seed vigor, phytohormone

## Abstract

Peanut (*Arachis hypogaea* L.) is an oilseed crop grown worldwide. Flavonoids have profound benefits for plant growth and development because of their powerful antioxidant properties. Seed vigor is an important indicator of seed quality. However, how flavonoids impact seed vigor formation in large-seed peanuts is still poorly understood. Here, we profiled flavonoids, phytohormones, and transcriptomes of developing seeds of large-seed peanut varieties with low (ZP06) and high (H8107) seed vigor. A total of 165 flavonoids were identified, 51 of which were differentially accumulated in ZP06 and H8107. Lower levels of dihydromyricetin (0.28 times) and hesperetin-7-O-glucoside (0.26 times) were observed in ZP06 seeds than in H8107. All flavonoid biosynthesis structural genes were down-regulated in ZP06. The different hormone levels found in ZP06 and H8107 seeds could be associated with the expression of flavonoid biosynthesis genes via MYB and bHLH transcription factors. Dihydromyricetin could relate to ZP06′s poor seed vigor by impacting its seed antioxidant properties. Thus, the presence of flavonoids in large-seed peanuts could contribute to their physiological quality and germination potential through controlling the accumulation of reactive oxygen species to improve seed antioxidant properties.

## 1. Introduction

Peanuts (*Arachis hypogaea* L.) are an important oilseed crop grown worldwide, with significant importance in agriculture, food security, and human health. The seed is the most important part in agricultural production. Large seeds usually contain more nutrients and energy, and thus have a higher germination potential. Seed size also affects seedling morphogenesis and establishment, with seedlings germinating from larger seeds often showing a stronger environmental-stress tolerance [1]. Taken together, these seed-related traits ultimately impact yield. Thus, developing peanut varieties with large seeds is an important goal.

Another important trait affecting germination and seedling development is seed vigor, which refers to the overall potential of seeds to rapidly and uniformly emerge and develop into seedlings under various field conditions. This trait is closely associated with multiple processes, such as seed development, maturation, dormancy, germination, seedling establishment, and stress tolerance; it also affects the quality of seeds during storage [2]. Seed vigor is largely determined by genes, and differs between plant varieties or strains. For example, studies have shown significant differences in seed vigor indicators among different genotypes of Arabidopsis [3], rice [4], wheat [5], and maize [6]. The development of metabolomic, transcriptomic, and proteomic technologies has enabled large-scale analysis of the key genes and proteins underlying such differences [6]. So far, no genes related to peanut seed vigor-related traits have been cloned.

Flavonoids are secondary metabolites that play an important role in multiple plant growth and developmental processes. It was reported that flavonoids were shown to be indispensable for complete male fertility in rice [7]. In grape, enhancement of the total flavonoid content through exogenous indoleacetic acid (IAA)-mediated increases in chalcone synthase (CHS), which catalyzes the first step in flavonoid biosynthesis, positively affects fruit quality [8]. The maturation process of strawberry is closely related to flavonoids [9]. Flavonoids also accumulate during seed formation. The major types of flavonoids in seeds are flavonols, anthocyanins, phlobaphenes, isoflavones, and proanthocyanidins [10]. The specific types of flavonoids present in seeds differ among plant species, but seeds of both monocotyledonous and dicotyledonous plants accumulate proanthocyanidins [11]. Flavonoids affect the color of the seed coat, and they may also be involved in controlling the development of the seed coat, the endosperm, fertilization, and the embryo, as well as signal transduction among them, and the regulation of seed dormancy and vigor [10]. Quercetin, a special subclass of flavonoid [12], was reported to improve the germination percentage and seed vigor in *Apocynum venetum* and *Apocynum pictum* under mannitol-induced osmotic stress [13]. Moreover, citrus bioflavonoids were found to enhance canola and soybean seed germination under both ideal and stressful conditions [14]. The relationship between flavonoids, antioxidant activity, and seed germination is complex.

In addition, seed vigor is regulated by the coordinated action of hormone metabolism pathways such as the abscisic acid (ABA) pathway, which plays an important role in seed development and germination [15] by affecting the accumulation of storage proteins and lipids, the acquisition of dehydration tolerance, and the induction and maintenance of seed dormancy [16,17]. For example, *ZmABA8ox*, which encodes a key enzyme involved in ABA degradation, is highly expressed in the high-vigor maize hybrid variety B73/Mo17, and genetic transformation experiments in Arabidopsis confirmed that increasing the expression level of this gene promotes seed germination [18]. Seed vigor may also be regulated by interactions between hormones. For example, the indole-3-acetate beta-glucosyltransferase gene *OsIAGLU* was found to regulate seed vigor by mediating crosstalk between auxin and ABA in rice [19].

For now, the specific molecular mechanisms by which flavonoids affect seed vigor are still unclear and require further study. Thus, in this study, we performed a metabolomic analysis of the flavonoid metabolites in seeds of large-seed peanut lines with different degrees of seed vigor. In addition, we explored the molecular mechanism underlying the effect of flavonoids on the vigor of large-seed peanut varieties by performing combined analysis of the metabolome and transcriptome data.

## 2. Materials and Methods

### 2.1. Plant Materials and Germination Experiments

The peanut sib line cultivars ZP06 and H8107, bred at Henan Agricultural University, were used as materials. Both ZP06 and H8107 were grown in the scientific and educational park of Henan Agricultural University, Zhengzhou City, China (113.63E, 37.75N), which has the features of a temperate continental monsoon climate, tidal soil, and moderate soil acidity and alkalinity. The days to first flower are similar for both of them: approximately 30 days after sowing. Pod samples for further experiments were collected at specific developmental stages, including 25, 35, 45, 55, and 65 days after flowering. Pods were manually separated into shells and seeds. All shells and seeds for each cultivar at developmental stages were mixed, frozen in liquid nitrogen immediately, and stored at −80 °C for further experimentation. The content levels of oleic acid, linoleic acid, oil, and protein of ZP06 and H8107 were measured according to the methods in Tang et al. [20].

Mature peanut seeds with uniform size, uniform plumpness, and no damage were selected for seed aging and subsequent germination tests. Naturally aged seeds had been stored for 6, 9, 12, 15, 18, and 21 months in dry and temperate conditions. Controlled deterioration treatment was performed as described below. Briefly, seeds were evenly put into an electronically controlled environment cabinet at 95% relative humidity (45 °C) for 0 (mature dry seeds, control), 1, 2, 3, 4, and 5 days, respectively. Then, the seeds were dried back to their moisture content before treatment at room temperature. For accelerated aging by methanol stress, firstly, the seeds were placed in a moist chamber at RT for 2 days to elevate seed moisture content. Then, the seeds were soaked in 50% (*v*/*v*) methanol for 0 (control), 15, 25, 35, 45, and 55 min, respectively. After the treatment, the seeds were dried with filter paper to remove the residual liquid on the surface, and then aired indoors for about 8 h before standby. For dihydromyricetin treatment, after methanol stress for 15 min, the seeds were soaked in a dihydromyricetin solution for 12 h, and then dried for 24 h. For the germination assay, 50 seeds from each treatment were sterilized with a 1% (*v*/*v*) solution of sodium hypochlorite for 1 min, and then the seeds were placed in an incubator at 28 °C for germination. Radicle length was observed and measured every day for seven days. Thus, the germination percentage was determined. A 2,3,5-triphenyltetrazolium chloride (TTC) reduction assay was conducted according to Gong et al. [21]. Superoxide dismutase (SOD) and peroxidase (POD) activity assays were conducted using detection reagent kits (KTB1030, Abbkine, and KTB1150, Abbkine, respectively).

### 2.2. Determination of Flavonoid Metabolomics

Six seed samples were selected and divided into two groups for the flavonoid metabolic study. Each group of samples has three biological replicates. Flavonoid metabolome profiling analysis was performed by Metware Biotechnology Co., Ltd. (Wuhan, China). The methods have been used previously in other studies [22]. In brief, seed samples were mixed and ground into powder. Around 100 mg seed powder was weighted and extracted overnight at 4 °C with 1 mL 70% methanol. After centrifugation, the extracts were absorbed, filtrated, and analyzed by LC-ESI-MS/MS. The detailed analytical conditions and equipment have been shown before [22]. Based on the Metware database and public database of metabolite information, qualitative analysis was conducted on the primary and secondary spectrum data from mass spectrometry detection. Some of these substances are qualitative. Isotopic signals, repeated signals containing K^+^, Na^+^, NH_4_^+^, and fragment ions of other substances with higher molecular weight, were removed during analysis. The analysis of metabolite structures was conducted with reference to the existing public mass spectrometry databases, including MassBank (http://www.massbank.jp/, accessed on 1 July 2024), KNAPSAcK (http://kanaya.naist.jp/KNApSAcK/, accessed on 1 July 2024), HMDB (http://www.hmdb.ca/, accessed on 1 July 2024), MoTo DB (http://www.ab.wur.nl/moto/, accessed on 1 July 2024), and METLIN (http://metlin.scripps.edu/index.php, accessed on 1 July 2024). Metabolite quantification was completed via multiple reaction monitoring analysis. After PCA and OPLS-DA analysis, the metabolites with |log2 (fold change)| ≥ 1 were selected for further analysis.

### 2.3. Determination of Phytohormones of Peanut Seeds

Phytohormone contents, including those of jasmonates (JAs), cytokinins (CKs), auxin, salicylic acid (SA), and ABA, were detected by MetWare Biotechnology Co., Ltd. (Wuhan, China) based on the AB Sciex QTRAP 6500 LC-MS/MS platform. Seeds (about 50 mg fresh weight) were ground into powder and extracted with methanol/formic acid/water (15:1:4, *v*/*v*/*v*). The combined extracts were evaporated to dryness under a nitrogen gas stream, reconstituted in 80% methanol (*v*/*v*), and filtrated (PTFE, 0.22 μm; Anpel, Shanghai, China) before LC-MS/MS analysis. The sample extracts were analyzed using an LC-ESI-MS/MS system (HPLC, Shim-pack UFLC SHIMADZU CBM30A system; MS, Applied Biosystems 6500 Triple Quadrupole). The analytical conditions were as follows: HPLC: column, Waters ACQUITY UPLC HSS T3 C18 (1.8 µm, 2.1 mm × 100 mm); solvent system, water (0.05% acetic acid): acetonitrile (0.05% acetic acid); gradient program, 95:5 *v*/*v* at 0 min, 95:5 *v*/*v* at 1 min, 5:95 *v*/*v* at 8 min, 5:95 *v*/*v* at 9 min, 95:5 *v*/*v* at 9.1 min, 95:5 *v*/*v* at 12 min; flow rate, 0.35 mL/min; temperature, 40 °C; injection volume: 2 μL. The effluent was alternatively connected to an ESI-triple quadrupole-linear ion trap (QTRAP)-MS. AB 6500 QTRAP LC/MS/MS System, equipped with an ESI Turbo Ion-Spray interface, operating in both positive and negative ion modes and controlled with Analyst 1.6 software (AB Sciex). The ESI source operation parameters were as follows: the ion source was turbo spray; the source temperature was 500 °C; the ion spray voltage (IS) was 4500 V; the curtain gas (CUR) was set at 35.0 psi; and the collision gas (CAD) was medium. DP and CE for individual MRM transitions were performed with further DP and CE optimization. A specific set of MRM transitions was monitored for each period according to the plant hormones eluted within this period.

### 2.4. RNA-Seq and Data Analysis

Total RNA was extracted from shells (K) and seeds (S), respectively, of ZP06 and H8107. The purity, concentration, and integrity of the RNA samples were tested using advanced molecular biology equipment to ensure the use of qualified samples for transcriptome sequencing. A total amount of 1 μg RNA per sample was used as input material for the RNA sample preparations. Sequencing libraries were generated using the NEBNext UltraTM RNA Library Prep Kit for Illumina (NEB, Ipswich, MA, USA) following the manufacturer’s recommendations, and index codes were added to attribute sequences to each sample. Clean data were obtained by removing reads containing adaptors, reads containing ploy-N, and low-quality reads from the raw data. Only clean reads with a perfect match or one mismatch to the reference genome sequence were further analyzed and annotated based on the reference genome. Genes with an adjusted *p*-value < 0.01 found by DEseq were assigned as differentially expressed. Gene function was annotated based on the following databases: Nr (NCBI non-redundant protein sequences), Nt (NCBI non-redundant nucleotide sequences), Pfam (Protein family), KOG/COG (Clusters of Orthologous Groups of proteins), Swiss-Prot (A manually annotated and reviewed protein sequence database), KO (KEGG Ortholog database), and GO (Gene Ontology).

### 2.5. Differential Expression of Genes, GO Enrichment, and KEGG Pathway Analyses

Since each seed sample from H1314 and H2014 has two biological replicates at S1 and S4, identification of differentially expressed genes (DEGs) between the mutant and the wild type was performed using the DESeq2 R package (1.20.0), and those genes with an adjusted *p*-value < 0.05 were assigned as differentially expressed. Enrichment analyses of GO terms and the KEGG pathway were carried out by using the GOseq R package based on the Wallenius non-central hyper-geometric distribution and KOBAS, respectively.

### 2.6. Integrated Analysis Between Transcriptome Data and Metabolites

Transcriptomic and metabolomic data, including on phytohormones and metabolites of the flavonoid pathway, were uniformly normalized by log2 transformation. PCA was carried out on transcriptomes and metabolomes, respectively, to visually show whether there are differences between the sample groups in transcriptomes and metabolomes, respectively. Correlation analysis was performed on the genes and metabolites detected in each different group. The Pearson correlation coefficient of genes and metabolites was calculated using the cor program in R language, and the difference times of gene metabolites with Pearson correlation coefficients greater than 0.8 in each different group were displayed. The correlation analysis of different genes and metabolites was carried out, and the results with Pearson correlation coefficients greater than 0.8 were finally selected.

### 2.7. qRT-PCR Analysis

Total RNA was extracted from peanut shells and seeds of H8107 and ZP06 using TRIzol (Invitrogen, Waltham, MA, USA) according to the manufacturer’s instructions. Quantitative real-time PCR analysis was carried out using TB Green Premix Ex Taq II (Tli RNaseH Plus) mix (TaKaRa) on a CFX96 Touch Real-Time PCR System (Bio-Rad, Hercules, CA, USA). For each gene, three repeats of each reaction were performed, and the relative expression was calculated by using the 2^−ΔΔCt^ method and normalized by using the internal reference actin gene. Thermal cycle parameters were 95 °C for 30 s, followed by 40 cycles of 95 °C for 10 s, and between 50 and 56 °C for 25 s in a 20 μL volume.

### 2.8. Statistical Analysis

One-way analysis of variance (ANOVA) with IBM SPSS statistical software 20.0 (SPSS Inc., Chicago, IL, USA) was used for statistical significance analysis. Data are presented as the mean ± standard error and/or standard deviation. For RNA-seq, differential expression analysis of two cultivars was performed using the DEGseq R package, and the *p*-value < 0.01 and |log_2_(foldchange)| ≥ 1 were set as the threshold for significantly differential expression. For flavonoid metabonomics, differentially accumulated metabolites of two cultivars were determined according to partial least squares-discriminant analysis, and the variable importance of the projection (VIP) ≥ 1 and |log_2_ fold change| ≥ 1 were defined as the threshold for significantly differential accumulation.

## 3. Results

### 3.1. Variation in Seed Vigor Between Peanut Varieties ZP06 and H8107

**Figure 1:**
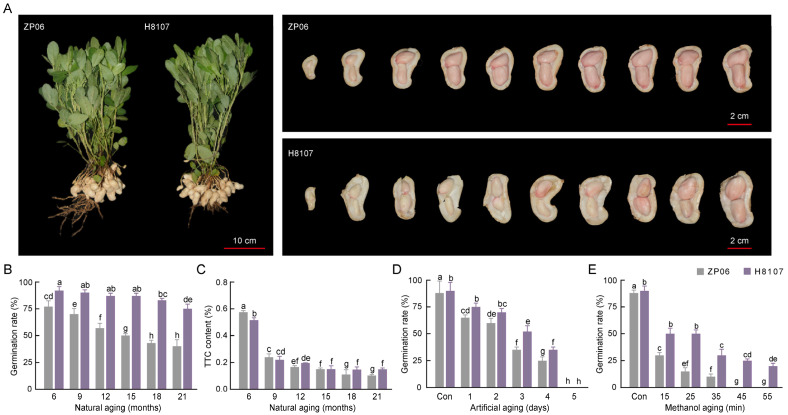
Phenotypic characterization of the peanut pods of ZP06 and H8107. (**A**) Plant and pod phenotypes. Pods were collected from a single plant. (**B**) Germination rates of ZP06 and H8107 seeds after natural aging for 6, 9, 12, 15, 18, and 21 months. (**C**) TTC contents of ZP06 and H8107 seeds after natural aging for 6, 9, 12, 15, 18, and 21 months. (**D**) Germination rates of ZP06 and H8107 seeds after artificial aging for 0, 1, 2, 3, 4, and 5 days. (**E**) Germination rates of ZP06 and H8107 seeds after methanol aging for 0, 15, 25, 35, 45, and 55 min. Different letters indicate significant differences (*p* < 0.05).

Peanut varieties ZP06 and H8107, which produce seeds with a red seed coat (also referred to as the seed skin and testa), have similar morphological and agronomic characteristics (Figure 1A). The pod and seed characteristics of ZP06 and H8107 were investigated, and the results showed that the pod of ZP06 was wider and thicker, while the pod length was about 3.52 mm shorter than that of H8107 (*p* < 0.05) (Figure S1A). There were more flat seeds for ZP06 (Figure S1B). Both varieties produce larger pods compared with other tetraploid cultivated species, including H103 and H7500 [23,24]. We measured the contents of oleic acid (Figure S1C), linoleic acid (Figure S1D), oil (Figure S1E), and protein (Figure S1F) in ZP06 and H8107 at different times during storage, and found no clear differences between varieties (Figure S1). We next investigated the seed vigor of ZP06 and H8107 by assessing germination after natural, artificial, and methanol aging. After natural aging for 6 months, the germination rate of ZP06 (72%) was lower than that of H8107 (91%) (Figure 1B). Although the germination rates of both varieties decreased with time, their difference in rates grew larger. After natural aging for 21 months, the germination rate of H8107 was 1.73 times that of ZP06. Correspondingly, the amount of TTC also decreased with aging time, and the TTC content in H8107 was generally higher than that in ZP06, especially after long-term aging (Figure 1C). Harvested seeds were used for artificial aging and methanol aging experiments. The germination rates of unaged ZP06 and H8107 seeds were around 88% and 90%, respectively. After artificial aging for 3 days, the germination rates of ZP06 and H8107 decreased significantly to 35% and 52%, respectively, and after 5 days no seeds germinated (Figure 1D). After methanol aging for 45 min, no ZP06 seeds germinated, whereas 25% of H8107 seeds did (Figure 1E). On the whole, the germination rates of both genotypes decreased significantly after aging, and in general the germination rates of H8107 were higher than those of ZP06, indicating that the seed vigor of ZP06 was lower than that of H8107, even though both varieties produce large pods and seeds.

### 3.2. Flavonoid Metabolic Profiling and Differential Flavonoid Metabolite Analysis

**Figure 2:**
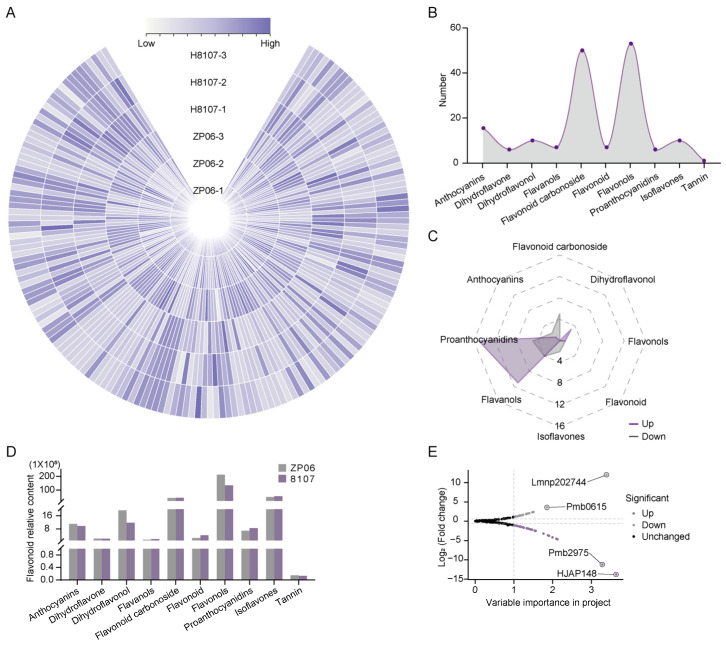
Flavonoid metabolite analysis of the large-seed peanut varieties ZP06 and H8107. (**A**) Circos plot showing the contents of all flavonoid types in each sample. Colors ranging from light blue to deep blue indicate an increase in content. (**B**) The numbers of each flavonoid metabolite. (**C**) Numbers of flavonoid metabolites showing differential accumulation between ZP06 and H8107. (**D**) The relative flavonoid metabolite contents in ZP06 and H8107. (**E**) Volcano plot showing differential accumulation of flavonoids. The flavonoids with the highest levels of differential accumulation are indicated.

H8107 and ZP06 seed samples were collected for flavonoid metabolomics analysis with LC-MS/MS (Figure S2A). Sample correlation analysis (Figure S2B) and unsupervised principal component analysis (Figure S2C) demonstrated consistency between biological replicates. A total of 165 flavonoid metabolites were detected in peanut seeds (Figure 2A, Table S1), namely 53 flavonols, 50 flavonoids, 15 anthocyanins, 10 dihydroflavonols, 10 proanthocyanidins, 7 flavonoid carbonosides, 7 flavanols, 6 dihydroflavones, 6 isoflavones, and 1 tannin (Figure 2B). In addition to being the most abundant in terms of number of types detected, flavonols were also the most abundant flavonoid metabolite in terms of content, with percent abundances of 61.46% in ZP06 and 49.78% in H8107, indicating that flavonols could be the main functional substances in peanuts. The next most abundant flavonoids were proanthocyanidins and flavonoids. In ZP06, the five flavonols with the highest contents were isohyperoside, procyanidin A1, quercetin-3-O-β-D-Galactoside (hyperin), quercetin-O-pentosyl-O-rhamnoside-O-glucoside, and quercetin-3-O-rutinoside (rutin).

Among all these metabolites, 51 were differentially accumulated in ZP06 and H8107; 31 were found at higher levels and 20 at lower levels in ZP06 seeds compared with H8107 seeds (Figure 2C, Table S2). The flavonoids with higher contents in ZP06 were 1 proanthocyanidin, 15 flavonols, 11 flavonoids, 1 dihydroflavonol, and 3 anthocyanins (Figure S2D). The five metabolites with the largest increases in ZP06 compared with H8107 were kaempferol-O-pentoside-O-hexoside (HJAP148, 14556.67 times), hesperetin O-glucuronic acid (pmb2975, 2437.48 times), kaempferol-3-O-robinobioside (pme1605, 26.24 times), cyanidin-3-O-(6′-p-coumaroylglucoside) (Lmpp003789, 23.42 times), and luteolin-7-O-rutinoside (pmp000593, 18.48 times) (Figure 2D,E). There was one proanthocyanidin, two isoflavones, five flavonols, five flavonoid carbonosides, four flavonoids, one flavanol, and two dihydroflavonols (Figure S2D) with lower abundance in ZP06, and the largest decreases were observed for three flavonoid carbonosides (Lmnp302744, Lmnp002494, and Lmnp002448), hesperetin C-hexosyl-O-hexosyl-O-hexoside (pmb0615, 0.08 times), and quercetin glu-glucuronic acid (Lmmp002560, 0.21 times) (Figure 2D,E). These identified differentially accumulated flavonoids were annotated using the KEGG database; the pathways included flavone and flavonol biosynthesis (ko00944), flavonoid biosynthesis (ko00941), biosynthesis of secondary metabolites (ko01110), anthocyanin biosynthesis (ko00942), and metabolic pathways (ko01100). Some of the modified flavonoid substances that we detected were not found in the KEGG database, and the related flavonoid metabolism pathway was constructed to supplement the existing KEGG, such as with naringenin-7-O-glucoside (mws1179) and eriodictyol C-hexoside (pmb3023) in flavonoid biosynthesis (ko00941 plus 1), and chrysoeriol-5-O-hexoside (pmb2999) in flavonoid biosynthesis (ko00941 plus 2).

### 3.3. Transcriptome Analysis and Identification of Differentially Expressed Genes Between H8107 and ZP06

Twelve cDNA libraries were prepared from the shells and seeds of H8107 and ZP06 and subjected to RNA sequencing (RNA-seq) analysis (Figure S3A). After removing adaptors and unknown or low-quality reads, 87,019,677 and 108,248,545 clean reads were obtained from shells, and 83,097,024 and 91,473,165 clean reads were obtained from seeds of H8107 and ZP06, respectively (Figure S3B). The Q30 of all libraries was >91.68%, and the GC content was approximately 46% (Table S3). Finally, 64,999 genes were identified (Table S4). To validate the RNA-seq data, we randomly chose 12 genes for qRT-PCR (Table S5), namely 1K8G8G, 0Y92P4, 4Y1607, AQ6B1J, 79B99S, 8F7PE4, LEZ1PQ, B1753N, K0UY8K, URN845, N3DIQ1, and QZN0LQ (Figure S3C).

**Figure 3:**
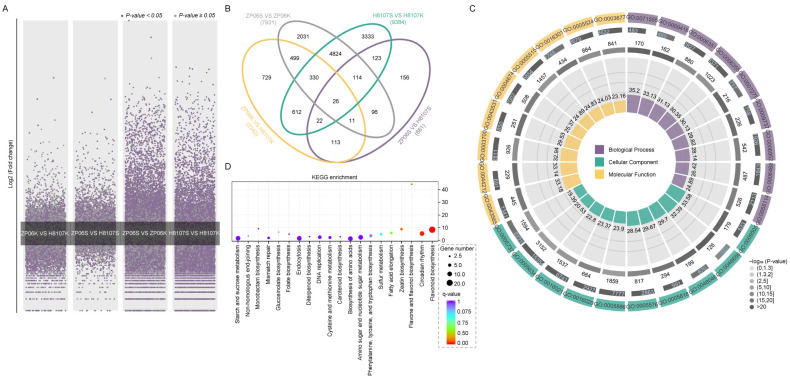
Transcriptomic analysis of the large-seed peanut cultivars ZP06 and H8107. (**A**) Chromosome gene coverage map of ZP06 and H8107. (**B**) Unique and shared DEGs between different comparison groups. (**C**) GO pathway analysis of DEGs in the seeds of ZP06 and H8107; details are shown in Table S11. (**D**) KEGG analysis of DEGs in the seeds of ZP06 and H8107.

We mapped all the identified genes to the cellular response pathway using MapMan and found that more genes were expressed in the shells than seeds and that these genes were involved in the biotic stress and abiotic stress pathway (Figure S4). Genes involved in development, cell division, and the cell cycle were expressed at higher levels in the seeds of both H8107 and ZP06 seeds than in the corresponding shells (Figure S4). DEGs were analyzed in each comparison group, and then all annotated gene sequences were matched against the COG, GO (Figure S5), KEGG (Figure S6), and KOG databases to predict possible functions. In total, there were 22,970 DEGs (Figure 3A, Table S6). When comparing the shell and seed of the same variety, there were 7931 DEGs identified for ZP06 (Table S7) and 9384 DEGs identified for H8107 (Table S8). Of these, 5294 DEGs were identified in both comparisons (Figure S7A), indicating that they are organ-specific genes (Figure S7B). KEGG analysis revealed that these organ-specific genes were highly enriched pathways including phenylpropanoid biosynthesis, plant hormone signal transduction, carbon metabolism, and starch and sucrose metabolism (Figure S7C). These results showed that the gene expression patterns of shells and seeds were obviously changed according to the tissues and their function.

Next, we compared gene expression between the seeds of ZP06 (ZP06S) and H8107 (H8107S) and identified 661 DEGs (Table S9, Figure 3B). Of these, 172 DEGs were also differentially expressed between the shells of ZP06 (ZP06K) and H8107 (H8107K) (Table S10, Figure S7D). These putative variety-specific DEGs (Figure S7E) were mainly involved in phenylpropanoid biosynthesis and amino sugar and nucleotide sugar metabolism (Figure S7F). Among all DEGs identified, those encoding a probable leucine-rich repeat receptor-like protein kinase (WE07RT), glycosyltransferase-like KOBITO 1 (PRAL1M), sulfite oxidase isoform X1 (JWY1N0), phosphoribulokinase (8L38IN), uncharacterized protein LOC107617222 (3V5M7L), and uncharacterized protein (1E7NTK) were only expressed in ZP06, and the transcription levels of DEGs encoding uncharacterized protein LOC107647108 (7PGW8X), uncharacterized protein LOC107470379 isoform X3 (5EE15R), and 1,2-dihydroxy-3-keto-5-methylthiopentene dioxygenase 2 (25NDHM) were very low in ZP06. Four genes encoding serine/threonine-protein kinase OXI1, BRI1-like 2, and ATG1a were lowly expressed in the developing seeds of ZP06 (Table S9). GO enrichment analysis of these DEGs revealed significant enrichment in 21 biological process terms, 16 cellular component terms, and 14 molecular function terms (Figure 3C, Table S11). The top enriched KEGG pathways were “flavonoid biosynthesis”, “plant hormone signal transduction”, “circadian rhythm-plant”, “biosynthesis of amino acids”, and “amino sugar and nucleotide sugar metabolism” (Figure 3D). In a subsequent analysis, we focused on the DEGs in the seed comparison group involved in these top KEGG pathways.

### 3.4. Differential Expression of Flavonoid Biosynthetic and Regulatory Genes During the Development of Peanut Seeds

**Figure 4:**
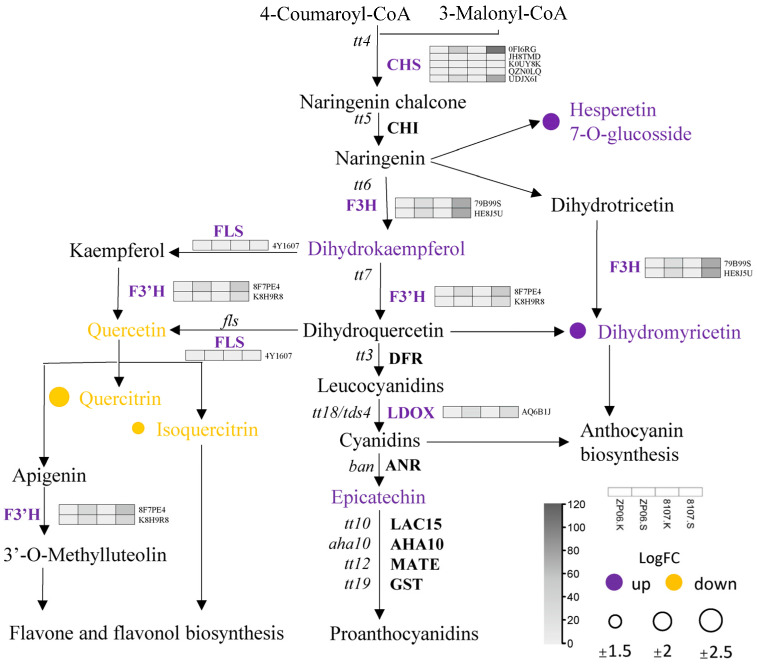
Expression profiles of DEGs associated with flavonoid biosynthesis in the seeds of ZP06 and H8107.

We next performed an integrated analysis of the transcriptome and flavonoid metabolomic data. In the flavonoid biosynthesis pathway, the first step is the conversion of 4-Coumaroyl-CoA and 3 Malonyl-CoA to naringenin chalcone by CHS, which is encoded by the *TRANSPARENT TESTA4* (*TT4*) gene. Next, naringenin is synthesized from naringenin chalcone by TT5, which is converted to flavonol by flavanone 3-hydroxylase (F3H), which is encoded by *TT6*, and flavonoid 3′-hydroxylase (F3′H), which is encoded by *TT7*. Synthesis of anthocyanins and proanthocyanidins is then catalyzed by a series of enzyme-encoding *TT* family genes and other genes (Figure 4). We found that the majority of transcripts related to the flavonoid biosynthetic pathway were down-regulated in ZP06, in particular genes encoding enzymes that converge in the production of dihydromyricetin, hesperetin-7-O-glucoside, dihydrokaempferol, and epicatechin. Specifically, 11 structural genes involved in flavonoid synthesis, transportation, and regulation were differentially expressed between ZP06 and H8107, namely *TT4* (0FI6RG, JH8TMD, K0UY8K, QZN0LQ, UDJX6I), *TT6* (79B99S, HE8J5U), *TT7* (8F7PE4, K8H9R8, 8F7PE4, K8H9R8), *TT18* (AQ6B1J), and *FLS* (encodes flavonol synthase; 4Y1607) (Table S9). All of them were down-regulated in ZP06 seeds compared with H8107 seeds. The corresponding flavonoid metabolites, including dihydromyricetin and hesperetin 7-O-glucosside, were also found to be down-regulated in ZP06 seeds (Table S2).

To investigate the transcription factors involved in the regulation of flavonoid synthesis, we analyzed members of the MYB and basic helix–loop–helix (bHLH) gene family. Five *R2R3MYB* genes and 10 *MYB*-related genes were detected. Among them, *M75BKX*, *EJIH9F*, and *WHG5AA* were significantly down-regulated in ZP06 seeds compared with H8107 seeds (Figure S8A, Table S6). A total of 273 bHLH family genes were detected and clustered into different groups: I (20), II (35), III (25), IV (10), V (32), VI (27), and VII (124). Twenty-eight of these genes were differentially expressed (nine down-regulated and nineteen up-regulated) in ZP06 seeds compared with H8107 seeds (Figure S8B, Table S6). For example, the expression of *D615WN* was one-sixth that in ZP06 seeds, whereas *577H6Y*, *UV35CQ*, and *HVT10G* were expressed at levels 14.6, 13.8, 12.6 times higher than those in H8107 seeds.

### 3.5. Phytohormone Contents and Conjoint Analysis with Transcriptome Analysis

**Figure 5:**
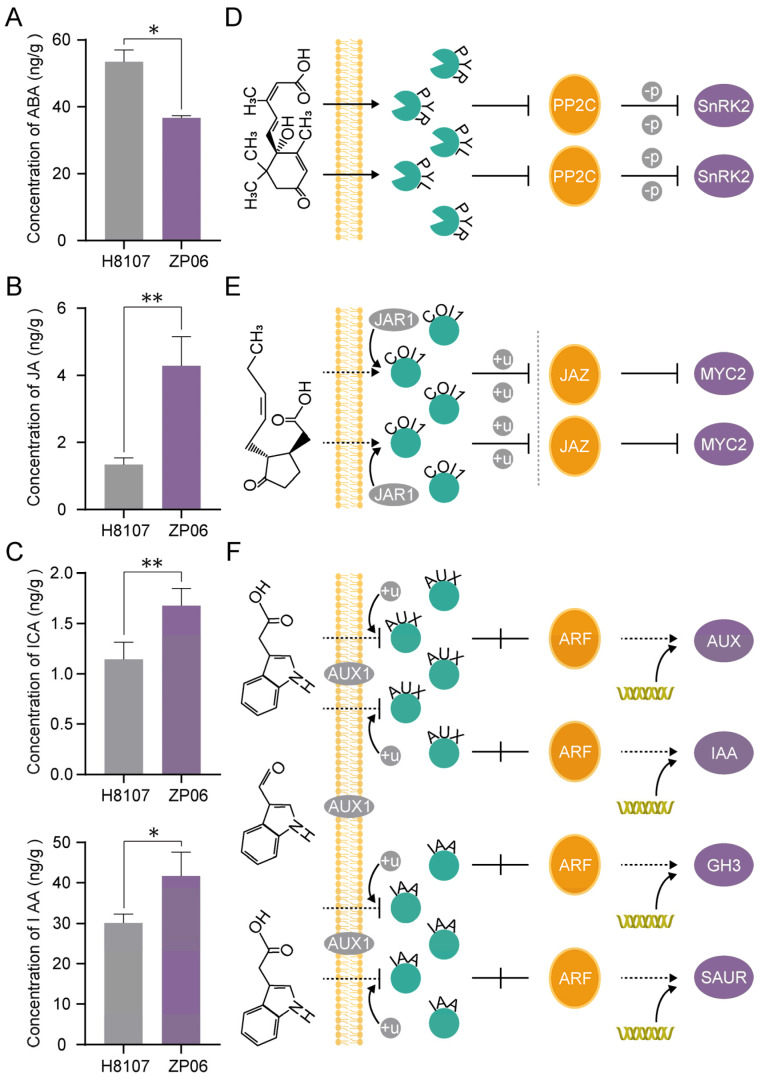
Analysis of hormones in the large-seed peanut varieties ZP06 and H8107. (**A**–**C**) The contents of ABA (**A**), JA (**B**), and ICA and IAA (**C**) in ZP06 and H8107 seeds. (**D**–**F**) DEGs involved in the ABA (**D**), JA (**E**), and IAA (**F**) signal pathways. “*” and “**” indicate significant differences at *p* < 0.05 and *p* < 0.01, respectively.

Transcriptome analysis of peanut seeds indicated that DEGs are enriched in the plant hormone signal transduction pathway. Thus, we conducted phytohormone analysis to quantitatively detect endogenous hormones in peanut seeds. Among the plant hormones, we failed to detect methyl jasmonate, *trans*-Zeatin, *cis*-Zeatin, and 3-indolebutyric acid by LC-MS/MS, possibly because of their low contents in seeds. Other hormones were successfully detected, and there were significant differences in the contents of ABA, JA, indole-3-carboxaldehyde (ICA), and IAA between ZP06 and H8107. The content of ABA was significantly lower (0.68 times) (Figure 5A), and the contents of JA (3.19 times) (Figure 5B), IAA (1.38 times), and ICA (1.47 times) (Figure 5C) were higher in the developing seeds of ZP06 than in those of H8107. After conjoint analysis with transcriptome data, we found that the ABA signal transduction pathway gene *SnRK2* (*LEZ1PQ*) was up-regulated in ZP06 seeds and had almost no expression in H8107 (Figure 5D), which was consistent with the lower content of ABA. The gene (*0Y92P4*) encoding ABA 8′-hydroxylase 3, which is involved in the oxidative degradation of ABA, was down-regulated in ZP06 seeds. Two *MYC2* genes (*D615WN* and *0RH9QK*) were differentially regulated in ZP06 seeds (Figure 5E). These genes both encode subgroup IV bHLH transcription factors, and MYC transcription factors such as these are important regulatory nodes of the JA signaling pathway. The expression of *D615WN* in ZP06 seeds was lower than that in H8107 seeds, while the expression pattern of *0RH9QK* was the opposite. Some *Aux/IAA* genes, which are part of the auxin signal transduction pathway (Figure 5F), were identified as being differentially expressed. The transcription levels of the *Aux/IAA* genes *FX782E* and *HJG3BW* were higher in ZP06 seeds than in H8107 seeds, while the transcription level of a gene (*newGene_3133*) encoding a putative auxin response factor 23 was lower. The genes *2G66YZ* and *VYJ22Y*, which encode the probable indole-3-acetic acid–amido synthetase GH3.1, were up-regulated in ZP06 seeds.

### 3.6. Dihydromyricetin Priming and Antioxidant System Activity of Peanut Seeds

**Figure 6:**
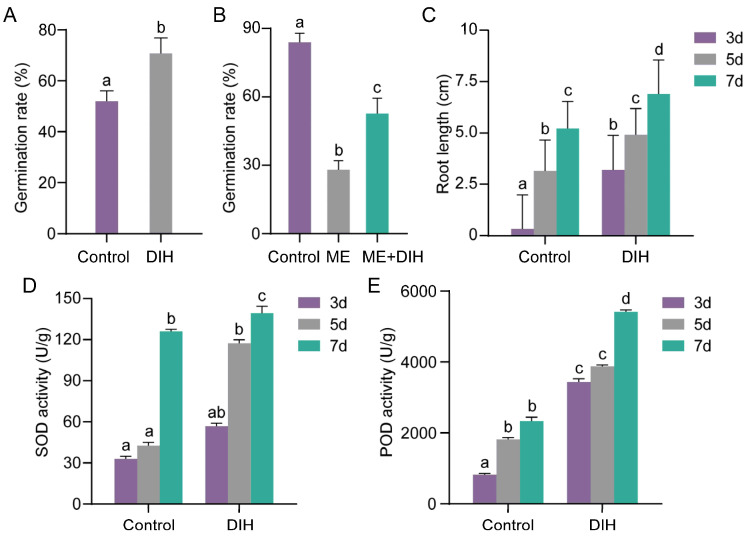
Dihydromyricetin priming treatment of peanut seeds. (**A**) Germination rates of ZP06 seeds after artificial aging (15 months) and dihydromyricetin treatment. Artificially aged ZP06 seeds (15 months) were used as a control. DIH, dihydromyricetin priming treatment. (**B**) Germination rates of ZP06 seeds after methanol aging (15 min) and dihydromyricetin treatment. Freshly harvested peanut seeds were used as a control. ME, methanol-aged seeds. ME + DIH, dihydromyricetin priming treatment carried out on methanol-aged seeds. (**C**–**E**) Root length (**C**), SOD enzyme activity (**D**), and POD enzyme activity for seedlings in (**A**). Different letters indicate significant differences (*p* < 0.05).

Dihydromyricetin is a flavonoid compound. In this study, there was less accumulation of dihydromyricetin in ZP06 seeds compared with H8107 seeds. We performed dihydromyricetin priming treatment on artificially and methanol-aged (15 min) ZP06 seeds to investigate its effect on seed vigor. We found that dihydromyricetin priming treatment increased the germination rates of artificially and methanol-aged seeds by 18.67% (Figure 6A) and 25.33% (Figure 6B), respectively. After treatment, there was also a significant increase in root length (Figure 6C). Thus, dihydromyricetin priming treatment improved ZP06 seed vigor. The enzyme activities of SOD (Figure 6D) and peroxidase POD (Figure 6E) in the treatment group were higher than those in the control group, indicating that dihydromyricetin treatment enhanced the enzymatic antioxidant capacity of ZP06 seeds.

### 3.7. Integrated Analysis of Differential Metabolites and DEGs in the Assembly Pathway Possibly Involved in Seed Vigor Formation

**Figure 7:**
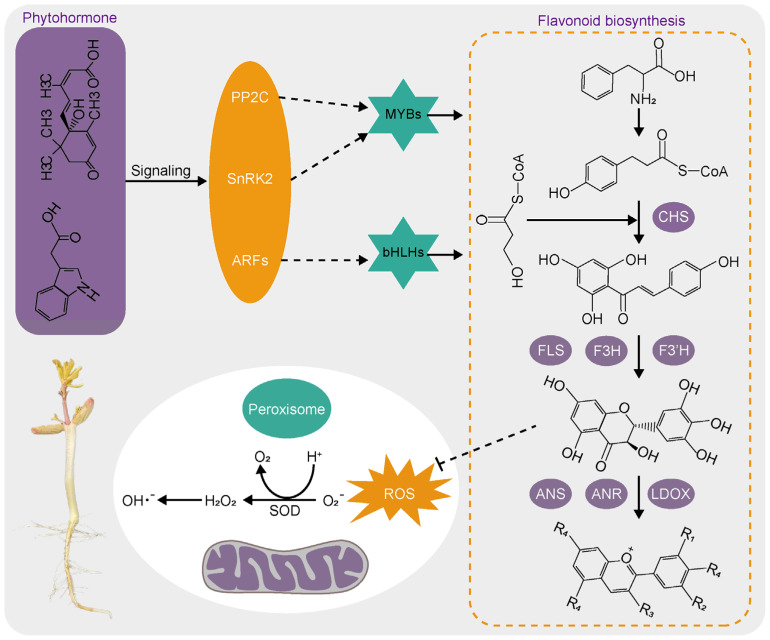
Putative phytohormone-regulated flavonoids involved in seed vigor formation.

Flavonoids play an important role in the growth and development of plants. Seed vigor is a complex trait, and it is mainly evaluated by monitoring germination. The vigor of seed germination is mainly determined by seed development. Plant flavonoids are synthesized by a series of structural enzymes, such as CHS, F3H, and FLS, which are regulated by transcription factors, including MYB and bHLH transcription factors. Flavonoid biosynthesis is modulated by hormones, whereby hormone levels affect the expression of genes and activities of key biosynthetic enzymes through the regulation of transcription factors, leading to variations in the synthesis of flavonoid metabolites between varieties (Figure 7). Differences in the accumulation of flavonoid metabolites result in disparities in seed vigor through the enzymatic antioxidant system acting on reactive oxygen species (Figure 7).

## 4. Discussion

In this study, we identified a total of 165 flavonoids from a metabolomics analysis of H8107 and ZP06 seeds at different developmental stages, without separating the peanut seed cotyledons and seed coat. For both cultivars, the metabolic category with the highest content was flavonols, followed by proanthocyanidins and flavonoids. The main forms of flavonols in ZP06 seeds were isohyperoside, hyperin, and rutin, and those in H8107 seeds were quercetin-7-O-(6′-O-malonyl)-β-D-glucoside, rutin, and isohyperoside. In addition, procyanidin A1 showed the highest amounts in both ZP06 and H8107, which could be mainly involved in seed coat color formation. Previous studies showed that type-A procyanidins predominate in peanut skins, while type-B procyanidins are rich in dietary sources [25,26,27]. We also found that there were far more type-A procyanidins (procyanidin A1 and A2) than type-B procyanidins (procyanidin B1, B2, B3 and B4) in both ZP06 and H8107. Proanthocyanidins and kaempferol-3-O-rutinoside are the main flavonoids in the seed coats of peanut seeds with different colors [28]. Black peanut seed coats have high contents of multiple types of anthocyanins. In white peanut skin, flavonol metabolism dominates, and its main form is isorhamnetin-3-O-rutinoside [28]. Hence, flavonoids are present in both the embryo and seed coat of large-seed peanuts, and there are differences in the contents of components between the cotyledon and seed coat, which are related to their functions.

The contents of dihydromyricetin, hesperetin-7-O-glucoside, and other flavonoids in ZP06 seeds were lower than those in H8107 seeds. Correspondingly, flavonoid biosynthesis pathway-related genes were also down-regulated in ZP06 seeds (Figure 6). CHS is the first enzyme in the flavonoid pathway [29], and genes encoding downstream enzymes, including F3H, F3′H, FLS, and LDOX, were all down-regulated in ZP06 seeds. Thus, their low expression could contribute to the low accumulation of dihydromyricetin, hesperetin-7-O-glucoside, and other flavonoids in ZP06 seeds. It was reported that the *CHS* RNAi transgenic tomato displayed impaired pollen tube growth and produced parthenocarpic fruits [30]. The rice *osf3h* CRISPR/Cas9 mutants displayed reduced seed yield, indicating that flavonoids are essential for complete male fertility in rice [12]. These above results suggest that in large-seed peanut varieties, the expression of genes such as *CHS* and *F3H* affects the differential accumulation of flavonoids, which regulate seed vitality by affecting seed development. By comparing germination indicators and antioxidant enzyme activities of seeds treated with dihydromyricetin and untreated seeds, we confirmed that dihydromyricetin has a positive effect on seed vigor. Treatment of peanut seeds with dihydromyricetin improved the antioxidant activity. This likely led to more effective clearing of reactive oxygen species and a reduction in fatty acid peroxidation, thereby improving peanut seed vigor.

Transcription factors (e.g., MYB-bHLH-WDR [MBW] complexes) regulate the expression of structural genes involved in flavonoid biosynthesis [31]. For example, the transcription levels of *CHS*, *F3H*, *DFR*, and *UF3GT* were higher in apple callus tissues overexpressing *MYC2* [32]. The transcription level of *AhMYC2* (D615WN) in ZP06 seeds was consistent with those of *AhCHS*, *AhF3H*, *AhF3′H*, *AhFLS*, and *AhLDOX*, and all of these genes showed decreased expression in ZP06 seeds compared with H8107 seeds, indicating that *AhMYC2* is involved in the control of flavonoid accumulation in peanut seeds. In addition, the transcription levels of *AhbHLH100* (H3TEND), *AhbHLH101* (H9FWK3), *AhMYB48* (EJIH9F), and *AhMYB25* (WHG5AA) in ZP06 seeds were lower than those in H8107 seeds, consistent with the expression patterns of structural genes involved in flavonoid biosynthesis. These results indicated that *AhbHLH100*, *AhbHLH101*, and *MYBs* could also play important roles in the regulation of flavonoid biosynthesis in peanuts through the regulation of structural gene expression and thereby impact various physiological processes such as growth, seed development, and seed vigor formation.

Flavonoids are related to hormone balance in seeds, particularly of auxin and ABA, which play a crucial role in seed germination and seedling development. High concentrations of quercetin, kaempferol, and their derivatives regulate root growth by limiting cell proliferation and accelerating cell elongation [33]. The mutant *tt7* exhibits inhibition of auxin transport [34], and the phenotype of this mutant is suppressed in the *ugt-78d2* and *rol1–2* mutants [35]. These findings indicated that quercetin, naringenin, and their glycosylated forms are the active forms of flavonols. Excessive accumulation of these forms can interfere with normal plant development [36]. Here, we found that the amounts of glycosylated forms of quercetin (namely quercetin-O-pentoside-O-hexoside-O-hexoside, hyperin, quercetin-3-O-β-D-glucoside, quercetin-O-hexoside-O-hexoside-O-pentoside, quercetin-O-rutinoside-hexose, quercetin-3-O-α-L-rhamnoside, quercetin-3-O-6′-O-malonyl-glucoside, and quercetin 3-O-β-D-xylopyranoside) and glycosylated forms of naringenin (namely 6-hydroxykaempferol-7-O-glucoside, 6-hydroxykaempferol-3, 6-O-diglucoside, and kaempferol-3-O-robinobioside) in ZP06 seeds were all higher than in H8107 seeds.

Although the auxin contents (including ICA and IAA) in ZP06 seeds were higher, their transport may be restricted by the excess flavonoids, thus resulting in disrupted seed development and poor seed vigor. On the other hand, it was reported that auxin can negatively regulate the expression of structural flavonoid biosynthesis genes, such as *CHS*, *F3H*, *MYB10*, and *PAP1-TT8/GL3-TTG1* [37]. AUX/IAA and ARF are important regulatory factors in the auxin signaling pathway. In apples, auxin-mediated inhibition of anthocyanin synthesis may occur through the MdIAA121–MdARF13 signal transduction pathway [38]. Here, we found that *Aux/IAA* (*FX782E* and *HJG3BW*) and *ARF23* (*newGene_3133*) were differentially expressed in ZP06 and H8107, and we speculate that the *AUX/IAA* gene (c52759) may play key roles in regulating anthocyanin biosynthesis in large-seed peanuts.

ABA promotes the biosynthesis of flavonoids, mainly anthocyanins and flavonols, by positively regulating the expression of the MBW protein complex [12,39]. At the same time, ABA can enhance the expression of FLS and promote the biosynthesis of flavonols [40]. We found that the expression of ABA signaling-related genes, such as *SnRK2* (*LEZ1PQ*), was strongly correlated with the contents of anthocyanin and flavonols and the expression of genes (*FLS*, *DFR*, *ANS*) in the flavonoid pathway. Therefore, this gene may be involved in the regulation of anthocyanin and flavonol biosynthesis. A previous study found that up-regulation of *ZmABA8ox1b* contributes to seed germination heterosis by promoting cell expansion [10]. In this study, we found that the ABA content in ZP06 seeds was lower than that in H8107 seeds, as was the expression of *ABA 8′-hydroxylase 3*. This might be associated with cell expansion and also suboptimal seed dormancy of large-seed peanuts.

In summary, flavonoids are essential for plant protection against oxidative stress and play a role in seed germination and longevity. Antioxidant priming can be a useful technique to enhance seed viability, particularly in challenging storage conditions. The presence of flavonoids in seeds can contribute to their physiological quality and germination potential.

## 5. Conclusions

The present study shows that two large-seed peanut varieties had differences in seed vigor. Through targeted metabolomics, the composition and contents of flavonoids were analyzed, revealing that differentially accumulated flavonoids might be closely related to seed vigor. In particular, the contents of dihydromyricetin, hesperetin-7-O-glucoside, and other flavonoids in ZP06 seeds were also lower than in H8107 seeds. Via transcriptomic analysis, structural genes involved in flavonoid biosynthesis were discovered to all be down-regulated in ZP06. Moreover, the expression of DEGs, such as *MYC2*, *ABA 8′-hydroxylase 3*, *Aux*/*IAA*, and *bHLH100*, was correlated with differences in the amounts of ABA, auxin, and JA in ZP06. After the treatment with dihydromyricetin, the germination rates and root length were significantly improved, and the enzyme activities of SOD and peroxidase POD were also increased. Thus, it is speculated that the biosynthesis of flavonoids is regulated by hormones, and flavonoids play a crucial role in seed germination and seedling development, possibly by improving seed antioxidant properties. This study reveals the mechanism by which flavonoid synthesis affects seed vigor in large-seed peanuts, providing a theoretical basis for further exploring the molecular basis of peanut seed vigor and breeding high-vigor large-seed peanuts.

## Data Availability

The original contributions presented in this study are included in the article; further inquiries can be directed at the corresponding author.

## Supplementary Materials

The following supporting information can be downloaded at https://www.mdpi.com/article/10.3390/antiox13121497/s1: Figure S1: Characterization of the seed quality of the peanut varieties ZP06 and H8107; Figure S2: Qualitative and quantitative analysis of flavonoid metabolites of the peanut varieties ZP06 and H8107; Figure S3: Transcriptomic sequencing analysis; Figure S4: Mapping genes to the cellular response pathway using MapMan; Figure S5: GO term enrichment analysis of DEGs in the comparison groups ZP06K vs. ZP06S (A), H8107K vs. H8107S (B), ZP06S vs. H8107S (C), and ZP06K vs. H8107K (D); Figure S6: KEGG pathway enrichment analysis of DEGs in the comparison groups ZP06K vs. ZP06S (A), H8107K vs. H8107S (B), ZP06S vs. H8107S (C), and ZP06K vs. H8107K (D); Figure S7: Venn diagram showing the overlap in DEGs among the comparison groups; Figure S8: Phylogenetic analysis of *AhMYB* and *AhbHLH*; Table S1: Comprehensive information on all identified flavonoids in peanut varieties ZP06 and H8107; Table S2: Differentially accumulated flavonoids between ZP06 and H8107 seeds; Table S3: Overview of sequencing data; Table S4: Comprehensive transcriptome information for the seeds and shells of peanut varieties ZP06 and H8107; Table S5: Primer sequences used for qRT-PCR; Table S6: Differentially expressed genes between peanut varieties ZP06 and H8107; Table S7: Differentially expressed genes from the comparison group ZP06K vs. ZP06S; Table S8: Differentially expressed genes from the comparison group H8107K vs. H8107S; Table S9: Differentially expressed genes from the comparison group ZP06S vs. H8107S; Table S10: Differentially expressed genes from the comparison group ZP06K vs. H8107K; Table S11: Enriched gene ontology terms for DEGs in the seeds of peanut varieties ZP06 and H8107.
