# Supplementary figures and images for: Metabolomic and Transcriptomic Analysis Reveals Flavonoid-Mediated Regulation of Seed Antioxidant Properties in Peanut Seed Vigor
Source: Antioxidants (Basel). 2024 Dec 8;13(12):1497. doi: 10.3390/antiox13121497 (PMC11673639; doi:10.3390/antiox13121497)

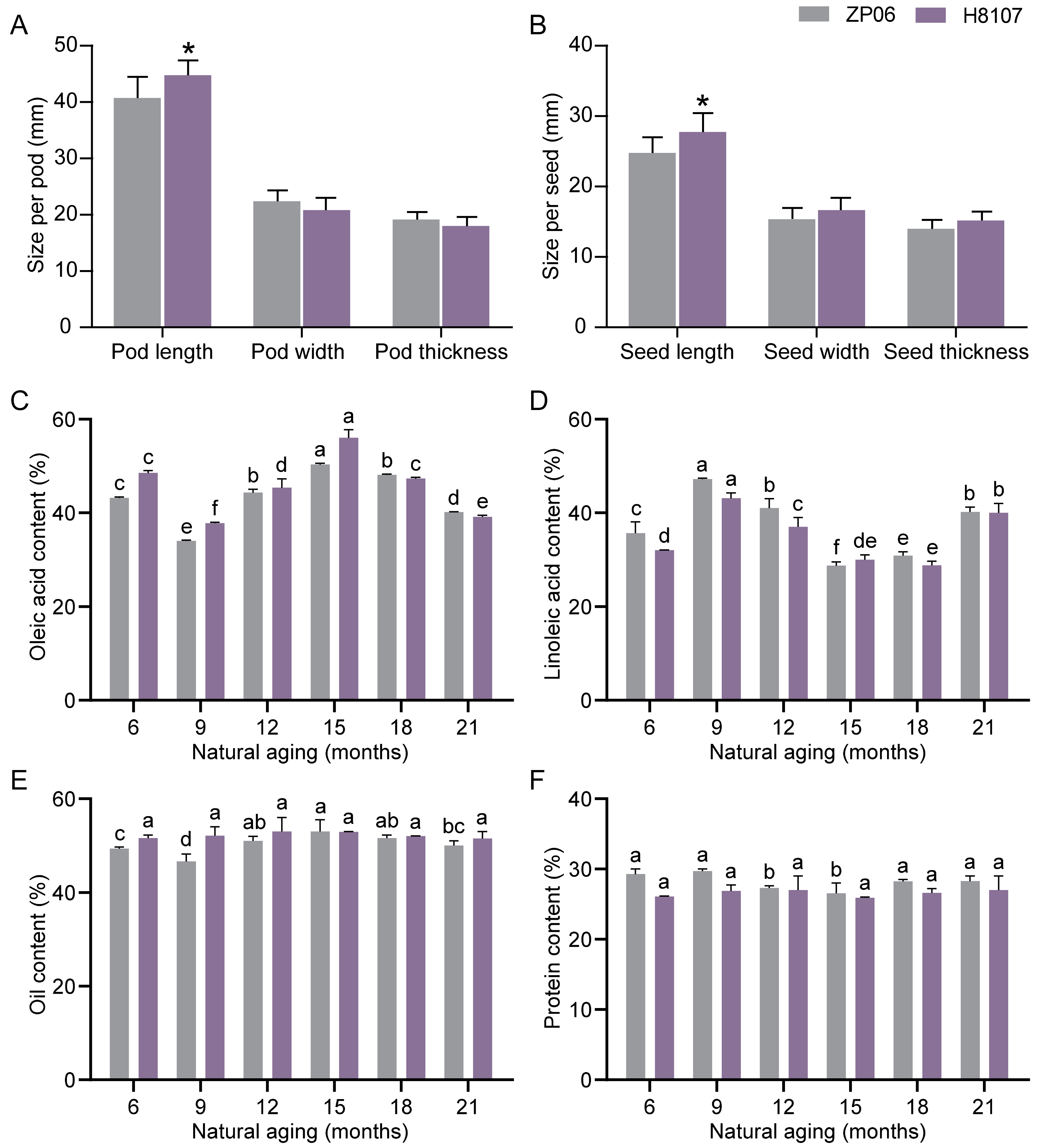

Supplement: Supplementary file 1 [file antioxidants-13-01497-s001.zip › Figure S1.tif]

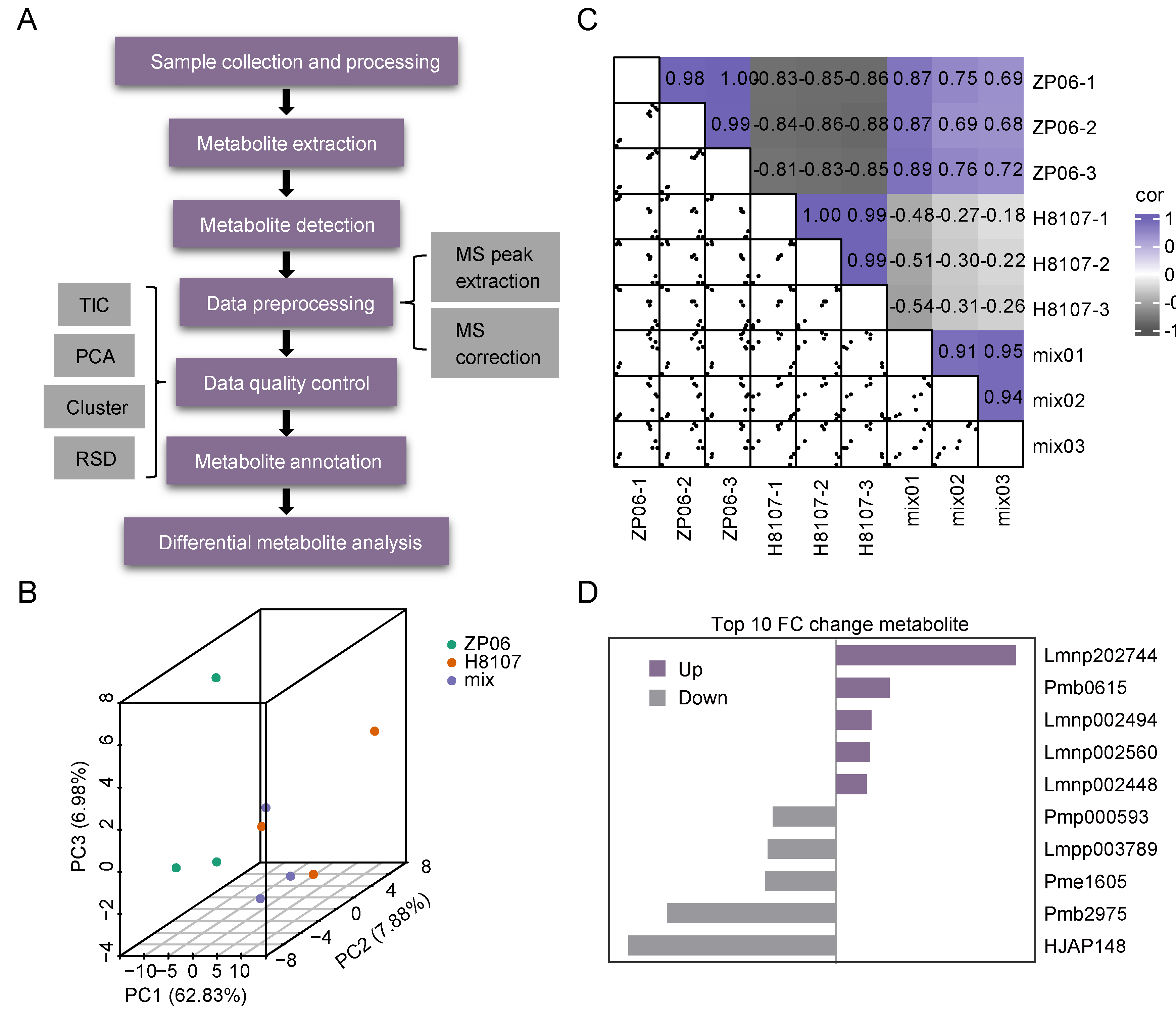

Supplement: Supplementary file 1 [file antioxidants-13-01497-s001.zip › Figure S2.tif]

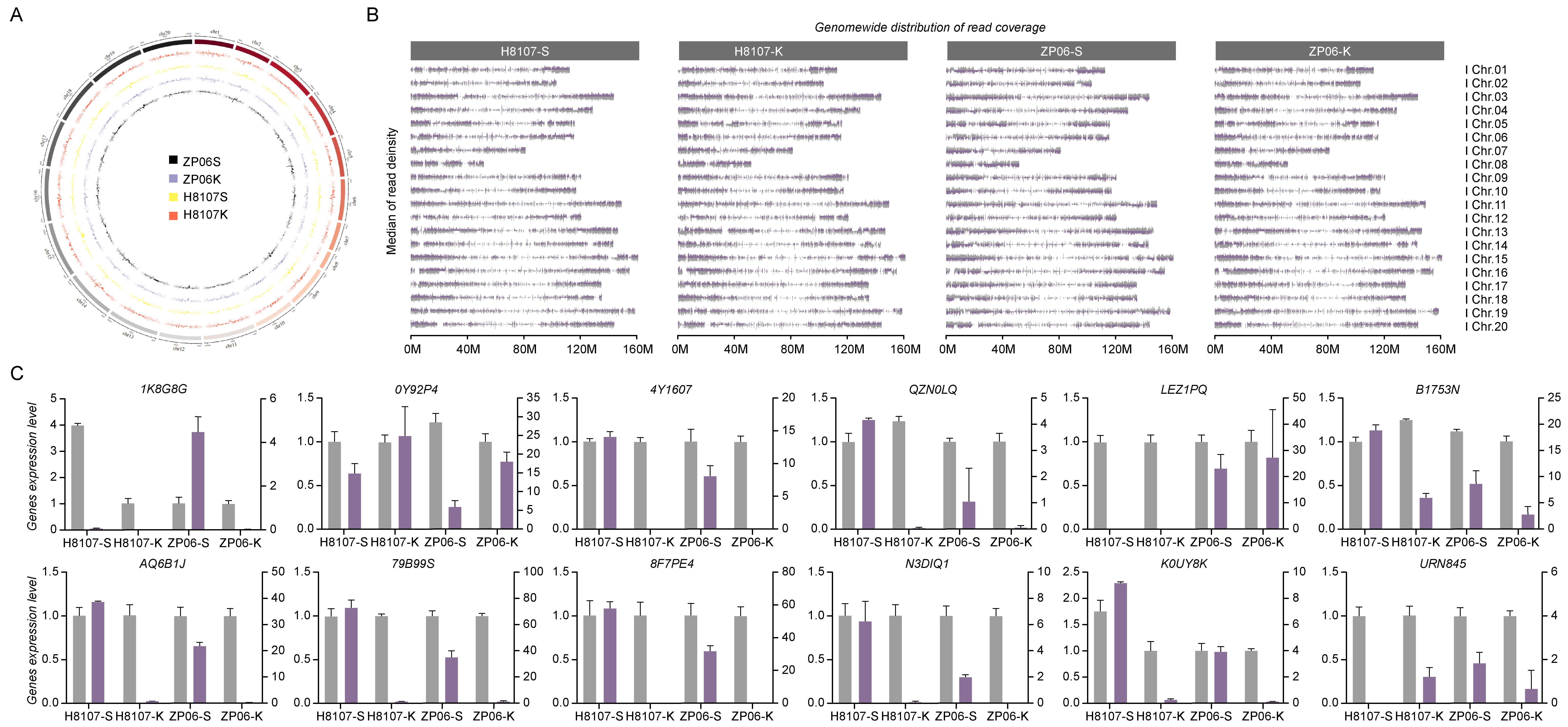

Supplement: Supplementary file 1 [file antioxidants-13-01497-s001.zip › Figure S3.tif]

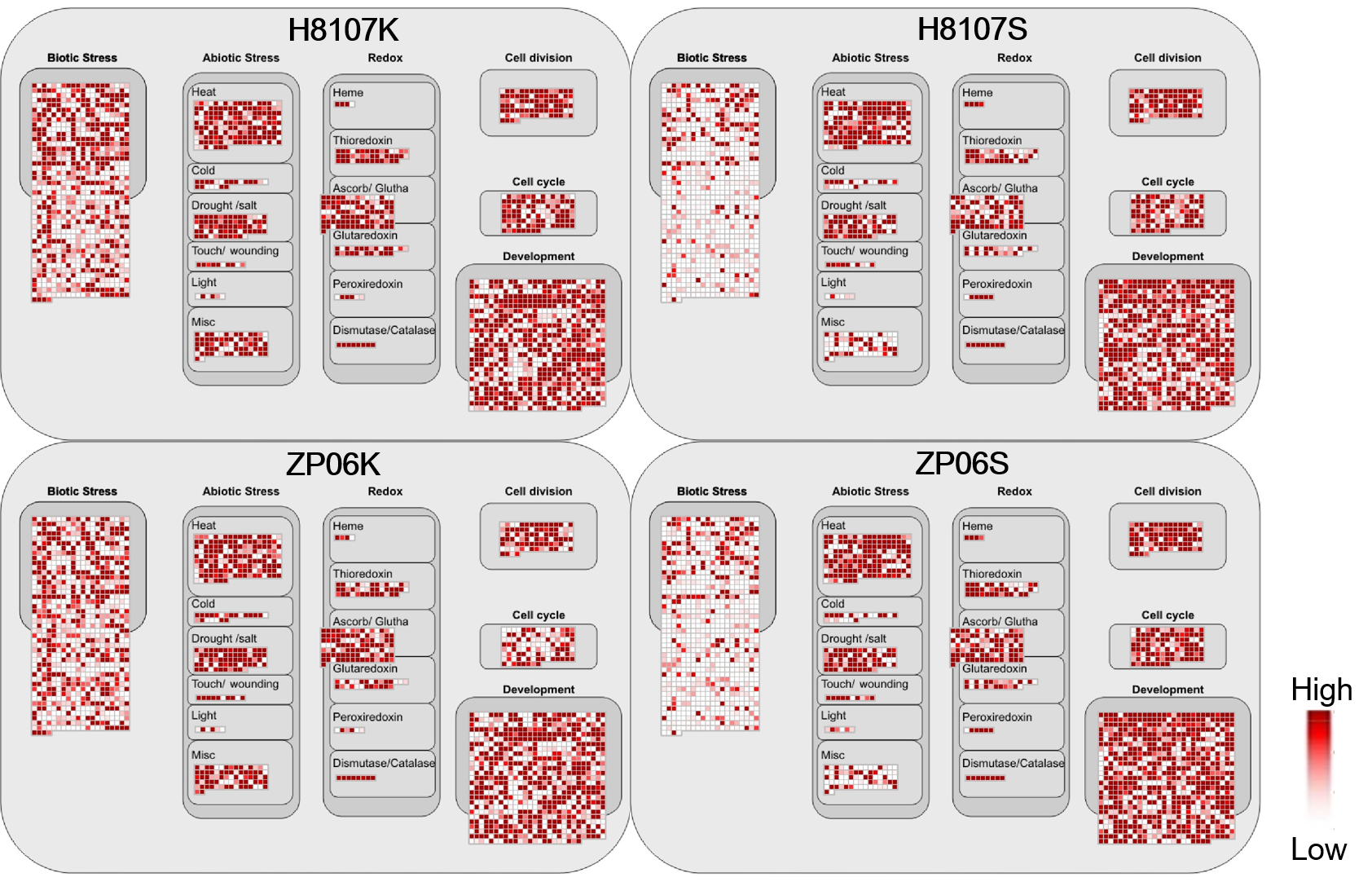

Supplement: Supplementary file 1 [file antioxidants-13-01497-s001.zip › Figure S4.tif]

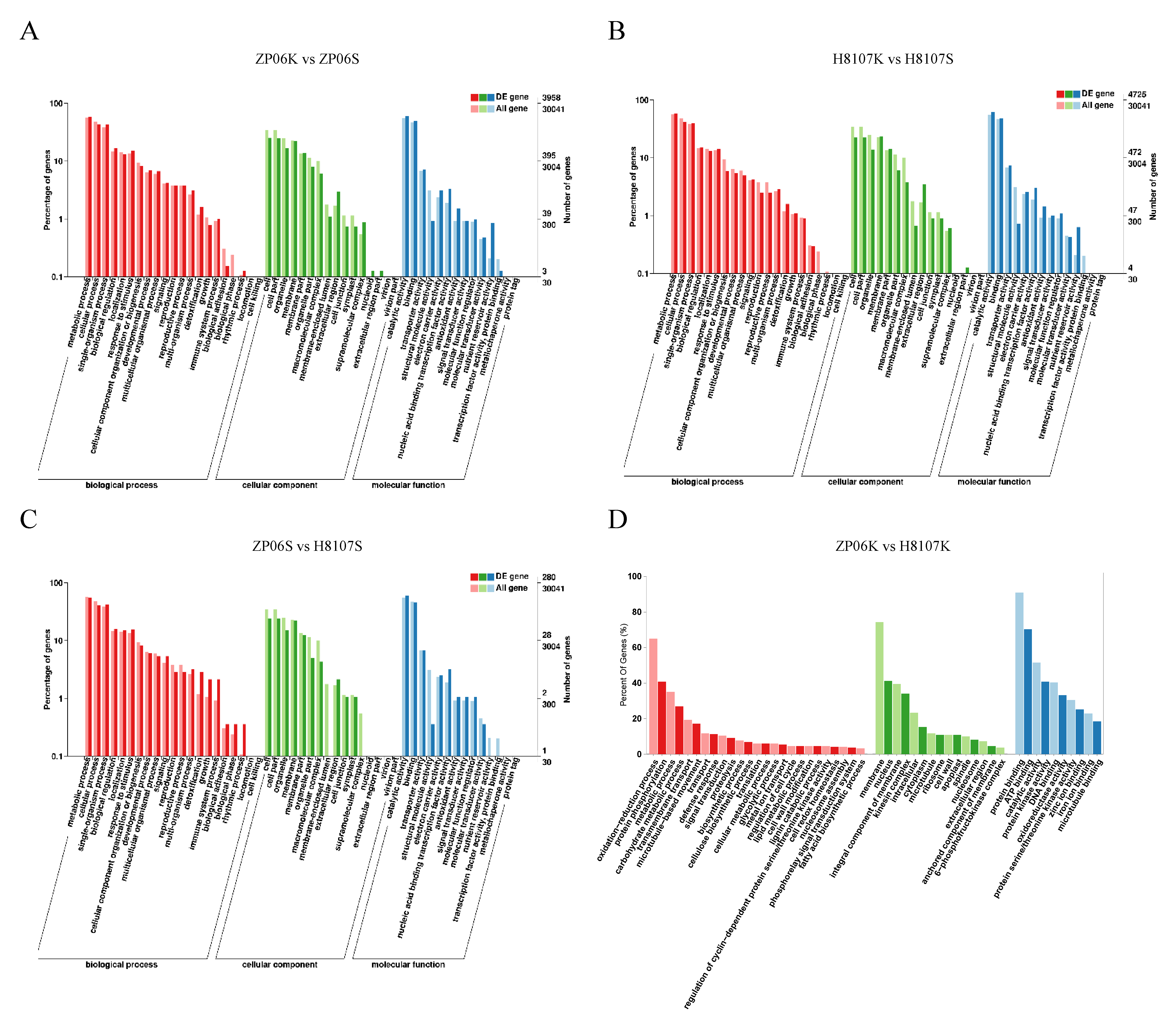

Supplement: Supplementary file 1 [file antioxidants-13-01497-s001.zip › Figure S5.tif]

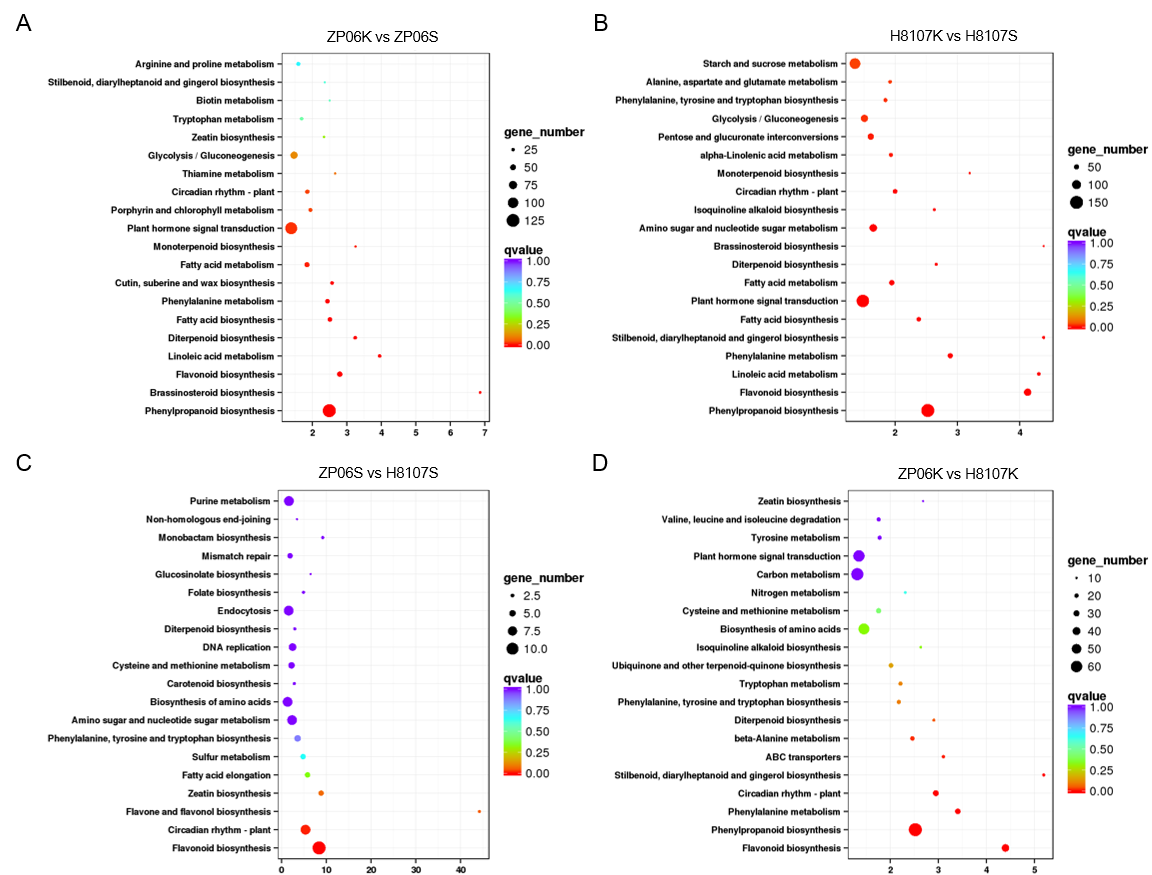

Supplement: Supplementary file 1 [file antioxidants-13-01497-s001.zip › Figure S6.tif]

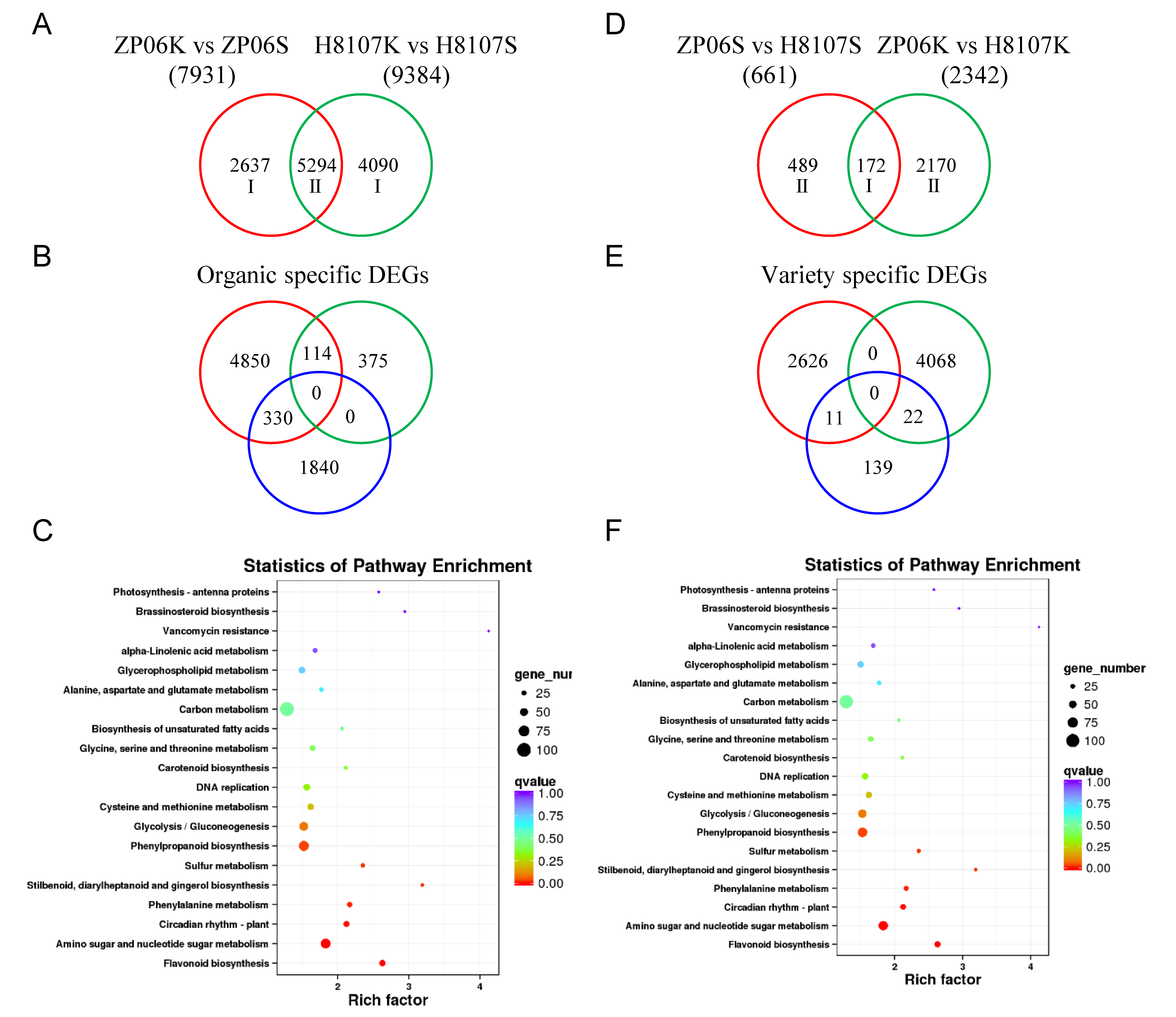

Supplement: Supplementary file 1 [file antioxidants-13-01497-s001.zip › Figure S7.tif]

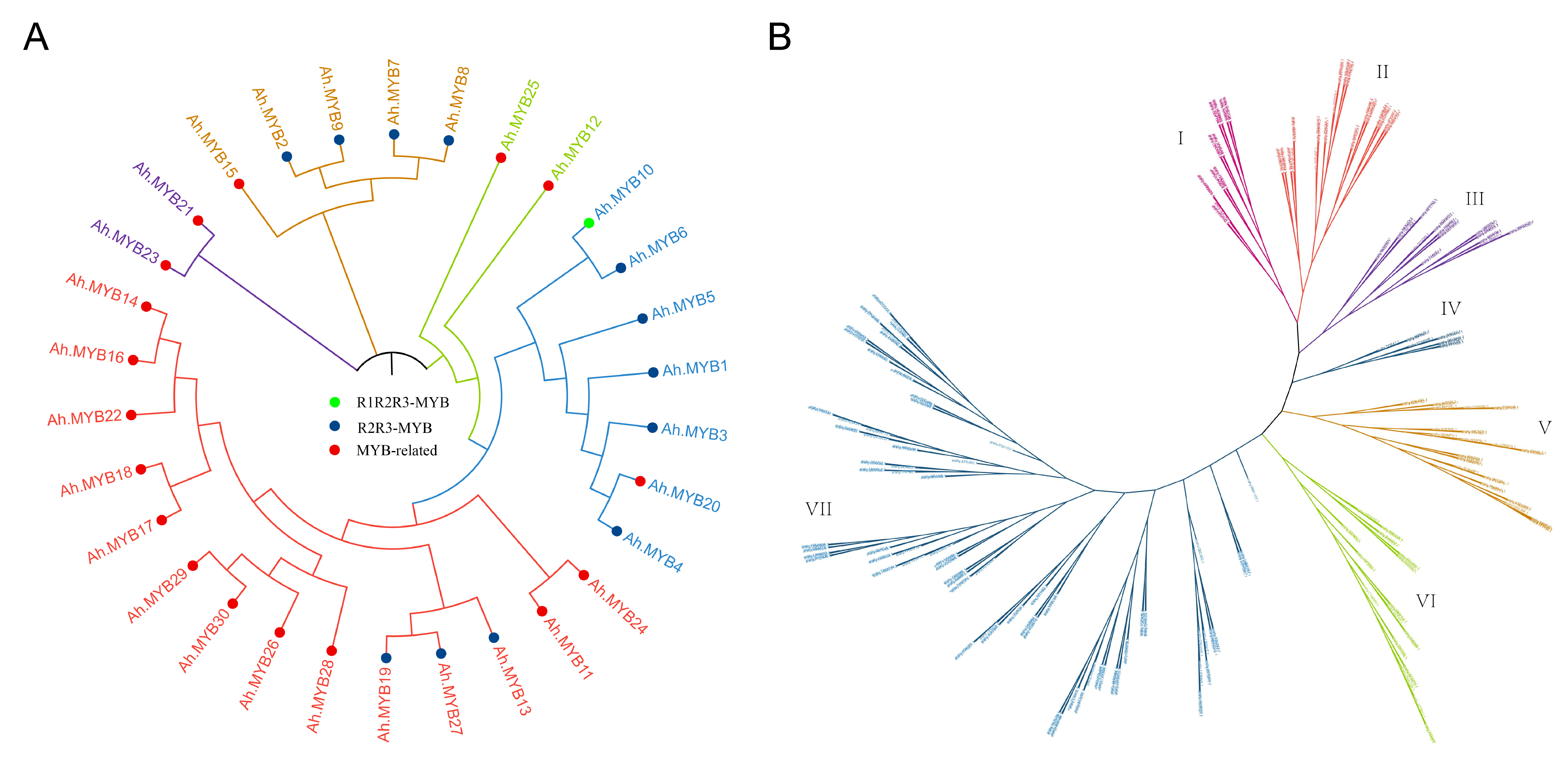

Supplement: Supplementary file 1 [file antioxidants-13-01497-s001.zip › Figure S8.tif]
